# LC-MS Analysis, 15-Lipoxygenase Inhibition, Cytotoxicity, and Genotoxicity of *Dissotis multiflora* (Sm) Triana (*Melastomataceae*) and *Paullinia pinnata* Linn (*Sapindaceae*)

**DOI:** 10.1155/2020/5169847

**Published:** 2020-02-18

**Authors:** Alian Désiré Afagnigni, Maximilienne Ascension Nyegue, Steve Valdi Djova, François-Xavier Etoa

**Affiliations:** ^1^Department of Biochemistry, Faculty of Science, University of Yaounde I, P.O. Box: 812, Yaounde, Cameroon; ^2^Department of Microbiology, Faculty of Science, University of Yaounde I, P.O. Box: 812, Yaounde, Cameroon

## Abstract

This study aims to evaluate the anti-inflammatory, cytotoxicity, and genotoxicity activities of *Dissotis multiflora* (Sm) Triana and *Paullinia pinnata* Linn used traditionally in Cameroon to treat infectious diseases. Phytochemical screening was carried out using the LC-MS procedure. The ferrous oxidation-xylenol orange (FOX) assay was used to determine the 15-lipoxygenase (15-LOX) inhibitory activity of the plant samples. The tetrazolium-based colorimetric (MTT) assay was performed using Vero cells. The Ames test was carried out using *Salmonella typhimurium* TA98 and TA100 tester strains. LC-MS chromatogram of *D. multiflora* led to the identification of four known compounds, namely, 5-(3,5-dinitrophenyl)-2H-tetrazol (2), 2,2'-{[2-(6-amino-9H-purine-9-yl)ethyl]imino}diethanol (14), 1,2,5-oxadiazolo [3,4-b]pyrazine, 5,6-di (3,5-dimethyl-1-piperidyl) (19), and nimbolinin D (20) while four compounds were also identified in *P. pinnata* known as 2-hydroxycarbamoyl-4-methyl-pentanoic acid (2), pheophorbide A (16), 1-[4-({2-[(1-methyl-1H-indol-5-yl)amino]-4-pyrimidinyl}oxy)-1-naphthyl]-3-[1-(4 methylphenyl)-3-(2-methyl-2-propanyl)-1H-pyrazol-5-yl]urea (17), and nimbolinin D (18). *D. multiflora* and *P. pinnata* inhibited 15-LOX activity in concentration-dependent manner. The LC_50_ (concentration that kills 50% of cells) values of the extracts ranged from 0.13 ± 00 to 1 ± 00 mg/mL for *P. pinnata* and *D. multiflora*, respectively. *P. pinnata* was cytotoxic at concentrations tested while *D. multiflora* was not. The selectivity index (SI) values ranged from 0.16 to 10.30 on Vero cell lines. No genotoxic effect was observed against both strains tested. These extracts are sources of compounds which can be used to control infectious diseases and associated inflammation. However, caution should be taken while using *P. pinnata* for medicinal purposes.

## 1. Background

Inflammation is the immune system's reaction to infection and injury involved in the pathogeneses of arthritis, cancer, stroke, neurodegenerative, and cardiovascular disease. This even removes offending factors and restores tissue structure and physiological function [1]. However, a massive production of proinflammatory molecules such as interleukin-6 (IL-6), tumour necrosis factor-*α* (TNF-*α*), and nitric oxide (NO) can modulate inflammation [2, 3].

Several studies have shown evidence of plant biological activities [4]. Since ancient times, plants are used for treatment of various diseases and are often assumed to be safe [5]. However, the lack of data on the toxicity of plants necessitates exhaustive evaluation of their safety given that some of them are a primary source of cytotoxic and genotoxic substances which can induce adverse effects [6–8]. Therefore, it is important to evaluate the efficacy and toxicity of natural products prior to potential use as antimicrobial agents [9].

In the search for effective safe natural antibacterial and anti-inflammatory compounds, the current study selected two Cameroonian medicinal plants based on their traditional use. Aqueous decoctions and powdered leaves from *Dissotis multiflora* (Sm) Triana from the Melastomataceae family are widely used in the Cameroon traditional medicine to treat infectious diseases and related conditions including diarrhea. In terms of biological activities, few studies have reported the presence of phytochemicals, antibacterial, antioxidant, and antidiarrheal activities of ethanolic leaf extract of *D. multiflora* [10, 11]. *Paullinia pinnata* Linn from the *Sapindaceae* family is used for the treatment of wounds and other skin diseases, typhoid, syphilis, gonorrhea, stomachache, waist pain, and diarrhea [12]. Modern pharmacology research has indicated that the different parts of *P. pinnata* possesses antioxidant, antidiarrheal, antityphoid, antibacterial, wound healing, cytoprotective, anti-inflammatory, and anxiolytic activities [13–19]. Phytochemical studies of *P. pinnata* revealed the presence of secondary metabolites while numerous phenolic compounds with biological activities have been isolated [20–24].

The widespread use of *D. multiflora* and *P. pinnata* for medicinal purposes to treat several infectious diseases motivated this study, knowing that there are no or few previous studies reporting their anti-inflammatory activity, cytotoxicity, and genotoxicity. Therefore, the present work was designed to investigate the 15-LOX inhibitory activity, cytotoxicity, and genotoxicity of *D. multiflora* and *P. pinnata* ethanolic extracts.

## 2. Materials and Methods

### 2.1. Plant Material and Extraction

The leaves of *Dissotis multiflora* (Sm) Triana (Melastomataceae) and *Paullinia pinnata* Linn (Sapindaceae) were collected in December 2013 in Nkoupa Matapit, West Cameroon. Plant identification and extraction were done as previously described [11, 19].

### 2.2. Cell Culture

African green monkey (Vero) kidney cell lines obtained from the American Type Culture Collection (ATCC) were maintained at 37°C and 5% CO_2_ in a humidified environment in modified Eagle's medium (MEM) high glucose (4.5 g/L) containing L-glutamine (Lonza, Belgium) and supplemented with 5% foetal bovine serum (Capricorn Scientific GmbH, South America) and 1% gentamicin (Virbac, RSA).

### 2.3. LC-MS Procedure

LC-MS analysis of *D. multiflora* and *P. pinnata* extracts was carried out following a modified method of Abay et al. [25] as previously described by Gheorghe et al. [26]. An oven with reverse phase column C_18_ (30°C) was used.

### 2.4. Inhibition of 15-Lipoxygenase (15-LOX) Enzyme

The assay was performed spectrophotometrically based on the formation of the complex Fe^3+^/xylenol orange according to Pinto et al. [27] as previously described by Motlhatlego et al. [28].

### 2.5. Cytotoxicity Assay

Cytotoxicity of the extract was determined in the MTT [3-(4, 5-dimethylthiazol-2-yl)-2, 5 diphenyltetrazolium bromide] reduction assay against Vero cell lines according to Mosmann [29] as described by Omokhua et al. [30]. From the previously reported MIC values [10, 19] and lethal concentration 50 (LC_50_) values obtained in the current study, the selectivity index (SI) values were calculated using the following formula: SI = LC_50_/MIC.

### 2.6. Genototoxicity Assay

The genotoxicity evaluation of plant samples was done in the histidine-deficient growth medium using the *Salmonella* microsome assay according to Maron and Ames [31] as described by Omokhua et al. [30].

### 2.7. Statistical Analysis

Each experiment was performed in triplicates. Data are expressed as mean ± standard deviation. Microsoft Excel was used to enter and capture data from which graphs and tables were extracted.

## 3. Results and Discussion

### 3.1. LC-MS Analysis of Plant Extracts

**Figure 1:**
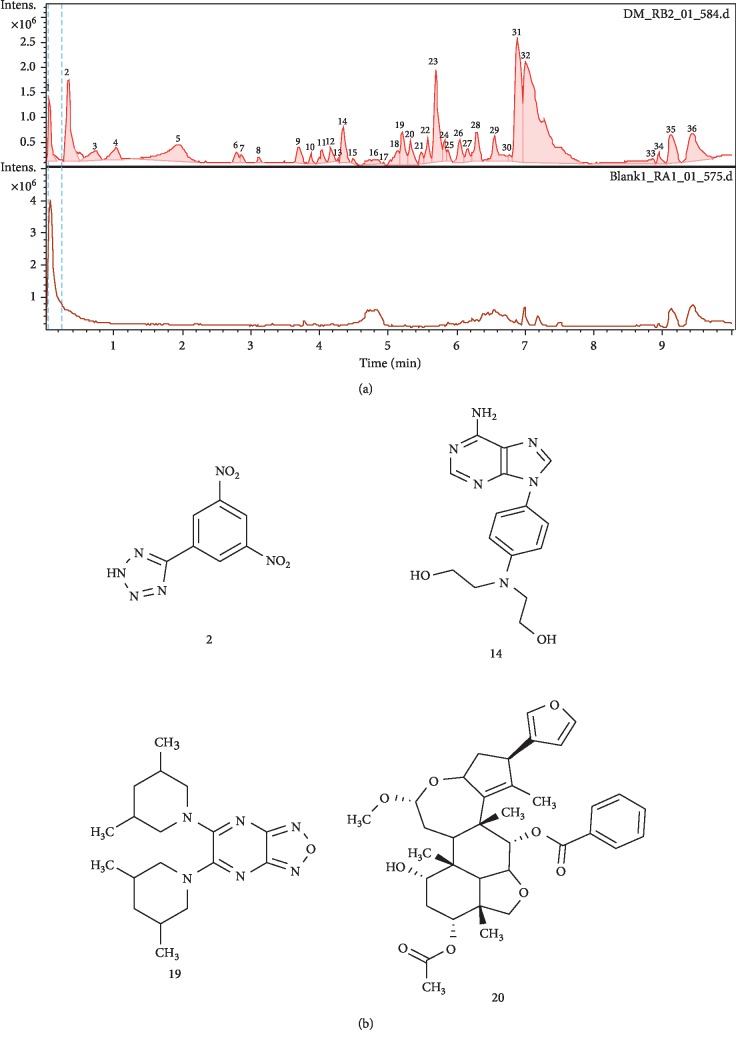
(a) LC-MS spectrum of *D. multiflora* leaf extract. (b) Chemical structures of 4 compounds identified in the leaf extract of *D. multiflora*.

LC-MS of *D. multiflora* and *P. pinnata* revealed the presence of many compounds with known or unknown therapeutic potentials. The LC-MS chromatogram of *D. multiflora* (Figure 1(a)) showed 36 peaks from which four compounds were identified (Figure 1(b)), namely, the 5-(3,5-dinitrophenyl)-2H-tetrazole (2), pseudo-molecular ion peak at m/z 290 g/mol [M + H]+ corresponding to the molecular formula C_7_H_4_N_6_O_4_ and retention time of 0.4 min, 2,2′-{[2-(6-amino-9H-purine-9-yl)ethyl]imino}diethanol (14) molecular formula C_15_H_18_N_6_O_2_, molecular weight 314.34 g/mol, and retention time of 4.4 min, 1,2,5-oxadiazolo [3,4-b]pyrazine, 5,6-di(3,5-dimethyl-1-piperidyl) (19), pseudo-molecular ion peak at m/z 345.2397 [M + H]+ corresponding to the molecular formula C_17_H_27_N_6_O and retention time of 5.2 min, and nimbolinin D (20), molecular formula C_36_H_44_O_9_, molecular ion peak at m/z 621.3083 g/mol [M + H]+, and retention time of 5.7 min. 5-(3,5-dinitrophenyl)-2H-tetrazole derivatives were found to possess antimycobacterial, antibacterial, antifungal, and cytotoxic properties [32] while nimbolinin D possess anti-inflammatory activity, especially the inhibition of the production of nitric oxide [33]. The presence of bioactive constituents in the ethanolic leaf of *D. multiflora* gives scientific basis for the use of this plant which is underexploited and here screened for the first time for its chromatographic profile.

**Figure 2:**
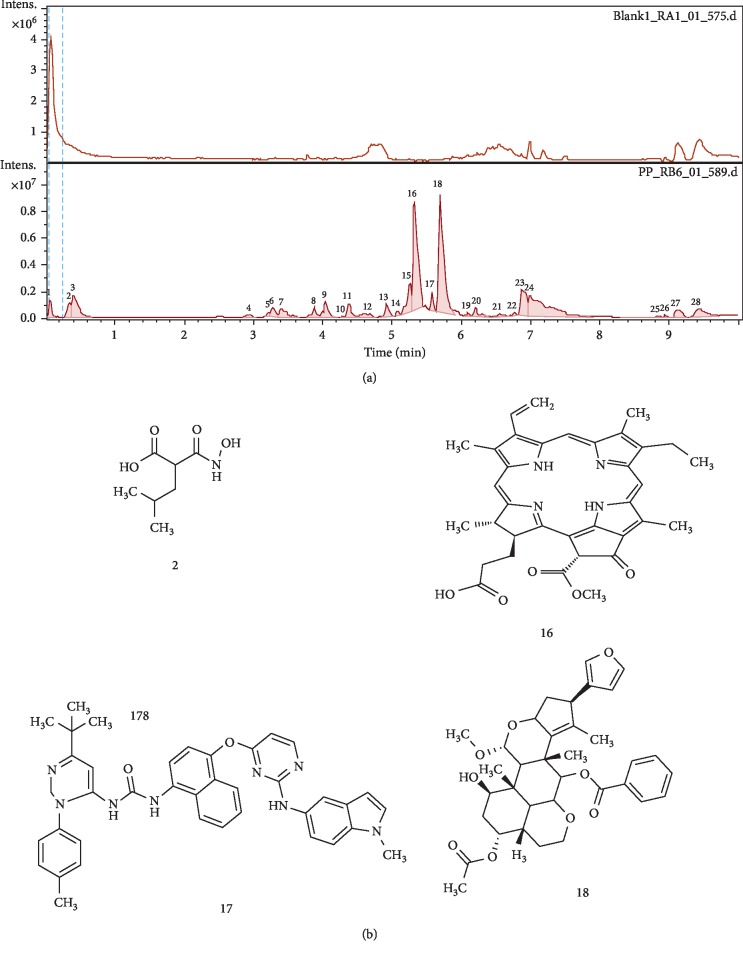
(a) LC-MS spectrum of *P. pinnata* leaf extract. (b) Chemical structures of 4 compounds identified in the leaf extract of *P. pinnata*.

Concerning *P*. *pinnata*, the LC-MS chromatogram (Figure 2(a)) showed 28 peaks from which four compounds were identified (Figure 2(b)), namely, the 2-hydroxycarbamoyl-4-methyl-pentanoic acid (2), molecular formula C_7_H_13_NO_4_, molecular weight 184 g/mol, and retention time of 0.4 min, pheophorbide A (16), molecular formula C_35_H_36_N_4_O_5_, molecular ion peak at m/z 593.2763 g/mol [M + H]+, and retention time of 5.4 min, 1-[4-({2-[(1-methyl-1H-indol-5-yl)amino]-4-pyrimidinyl}oxy)-1-naphthyl]-3-[1-(4 methylphenyl)-3-(2-methyl-2-propanyl)-1H-pyrazol-5-yl]urea (17), molecular formula C_37_H_36_N_8_O_2_, molecular weight 637.3042 g/mol, and retention time of 5.6 min, and nimbolinin (18) D, molecular formula C_36_H_44_O_9_, molecular ion peak at m/z 621.3083 g/mol [M + H]+, and retention time of 5.7 min. Amongst the fourth identified compounds, nimbolinin and pheophorbide A are known compounds present in other plants used also in traditional medicine. Nimbolinin D exhibited anti-inflammatory activity [33] while pheophorbide A was cytostatic, induced interruption of G0/G1 phasis and U87 MG cells apoptosis in the absence of direct photoactivation [34]. It also induced significant antiproliferative effects in a number of human cancer cell lines [35]. The identification of new constituents in *P. pinnata* leaf extract adds to the existing data that emphasize the presence of numerous bioactive compounds isolated and characterized [20–22, 24].

### 3.2. Inhibition of 15-Lipoxygenase (15-LOX) Enzyme

**Figure 3:**
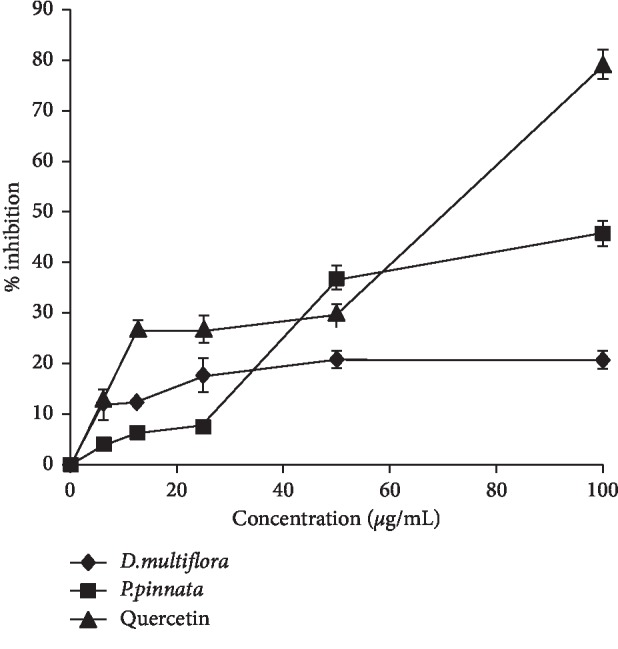
Inhibitory activity of the ethanolic extracts and fractions from *D. multiflora* and *P. pinnata* on 15-lipoxygenase. Data are expressed as mean ± standard deviation of three replicates.

The 15-LOX enzyme intervenes in the synthesis of leukotrienes from arachidonic acid. It has been proven that the bioactive leukotrienes are mediators of numerous proinflammatory and allergic reactions. Therefore, the 15-LOX inhibition activity is the most important one, even in the control of inflammatory conditions [36]. The 15-LOX inhibitory activity of ethanolic extracts from *D. multiflora* and *P. pinnata* was evaluated at different concentrations (6.25, 12.5, 25, 50, and 100 *μ*g/mL) and compared with quercetin (Figure 3). Extracts exhibited 15-LOX inhibitory effects in a concentration-dependant manner with inhibition percentage varying between 4 and 45.85% for the extracts of both plants. Phenol was found in both extracts and may be responsible of the 15-LOX inhibitory activity [10, 37].

### 3.3. Cytotoxicity Assay

The cytotoxicity test was performed against Vero monkey kidney cells to ensure the safe use of *D. multiflora* and *P. pinnata*. The LC_50_ values of the extracts ranged between 0.13 ± 00 and 1 ± 00 mg/mL for crude extracts from *P. pinnata* and *D. multiflora*, respectively (Table 1). It has been shown that LC_50_ values of 20 *μ*g/mL and below were considered to be toxic [38]. In this sense, all extracts are nontoxic. In a previous study, we determined the antibacterial activity of the ethanolic leaf extracts of *D. multiflora* and *P. pinnata* against *Salmonella typhi*, *Shigella flexneri*, *Proteus mirabilis*, *Klebsiella pneumoniae*, *Bacillus cereus*, and *Escherichia coli* [10, 19]. The SI values of the extracts were calculated by dividing the cytotoxicity LC_50_ (in mg/mL) by MIC (mg/mL). Results showed that the SI values ranged between 1.28 and 10.30 for *D. multiflora* while varying from 0.16 to 0.66 for *P. pinnata* (Table 1). According to Mongalo et al. [39], SI values above 1 refer to less toxic and below 1 to be toxic. Hence, *D. multiflora* with SI values above 1 showed no preliminary indication of toxicity while *P. pinnata* with SI values lower than 1 may be devoid of antibacterial activity. To the best of our knowledge, the present study reports for the first time the cytotoxicity of *D. multiflora*. In an *in vivo* study, *D. multiflora* was reported to be devoid of subacute toxicity at doses lower than 200 mg/kg. The cytotoxic potential of the crude plant extract of *Dissotis rotundifolia*, a plant of the same genera, was reported to be highly toxic to the MRC-5 cell line [40] while Abere et al. [41] reported that doses lower than 500 mg/kg were nontoxic by subacute toxicity. *P. pinnata* ethanolic leaf extracts were less active against the bacterial strains. Hence, the worse selectivity indices at concentrations tested. The *in vivo* toxicity test has shown that *P. pinnata* extract was not toxic at doses up to 200 mg/kg [24, 42]. One should observe that the results of *in vitro* toxicity evaluation differ substantially from that observed *in vivo*. This may be due to pharmacokinetic and pharmacodynamical considerations [43].

### 3.4. Genotoxicity Assay

The genotoxicity test was performed in order to determine ranges of plant extracts capable of producing genetic damage with resultant gene mutations without metabolic activation. From the Ames test, we observed that all the extracts tested at various concentrations had no number of revertant colonies of *S. typhimurium* strains TA98 and TA100 equal to or greater than twice those of the negative control (Table 2). Therefore, both plant extracts did not induce gene mutations [31]. In all cases, the values fall within normal limits and in accordance with the literature [44].

## 4. Conclusions

*D. multiflora* and *P. pinnata* extracts had 15-LOX inhibitory effects. The LC-MS led to the identification of compounds with known biological activities in both extracts. No cytotoxic effect was observed with *D. multiflora* and *P. pinnata* on the tested cell lines. However, *P. pinnata* is devoid of useful antibacterial activity justifying the worse selectivity indices. Our study indicated the potential nongenotoxic effect of both plant samples tested. However, study including a metabolic activation step is necessary to approve this finding. The information on 15-LOX inhibitory effects, cytotoxicity, and genotoxicity of these plant samples motivates further research that aims at isolating and determining biological properties of safe compounds from both plants.

## Figures and Tables

**Table 1:** Cytotoxic effects and selectivity index values of ethanolic leaf extract of *D. multiflora* and *P. pinnata* on Vero cells.

Plant	LC_50_ (mg/mL)	Selectivity index (SI)					
		*S. typhi*	*S. flex*	*K. pneu*	*B. cer*	*E. coli*	*P. mirab*
*D. multiflora*	1.00 ± 0.00	10.30	5.12	5.12	1.28	2.56	2.56
*P. pinnata*	0.13 ± 0.06	0.16	0.66	0.33	0.16	0.16	0.33
Doxorubicin	5.92 ± 1.21	—	—	—	—	—	—

*S. typhi*: *Salmonella typhi*; *S. flex*: *Shigella flexneri*; *K. pneu*: *K. pneumoniae*; *B. cer*: *Bacillus cereus*; *E. coli*: *Escherichia coli*; *P. mirab*: *Proteus mirabilis*. Doxorubicin hydrochloride in *μ*g/mL was used as positive control. Minimum inhibitory concentration values of extracts (mg/mL) from previous works; LC_50_ = Lowest concentration of extract which is lethal to 50% of the cells.

**Table 2:** Number of revertant colonies of *Salmonella typhimurium* TA98 and TA100 tester strains induced by ethanolic extracts of *D. multiflora* and *P*. *pinnata*.

Plant	Concentration (mg/mL)	His + revertants plate	
		TA98	TA100
*D. multiflora*	5	23.00 ± 0.57 (1.131)	135.00 ± 1.00 (1.298)
	0.5	21.66 ± 2.66 (1.065)	120.66 ± 2.33 (1.160)
	0.005	14.33 ± 0.33 (0.704)	133.00 ± 2.00 (1.278)

*P. pinnata*	5	19.00 ± 0.00 (0.934)	142.33 ± 2.33 (1.368)
	0.5	14.33 ± 0.33 (0.704)	139.33 ± 2.33 (1.339)
	0.005	7.33 ± 0.33 (0.360)	116.00 ± 0.00 (1.115)

4 NQO		128.00 ± 0.66 (2 *µ*g/mL)	389.33 ± 1.33 (1 *µ*g/mL)

10% DMSO		22.00 ± 0.57 (1.082)	98.12 ± 0.33 (0.919)

Water		20.33 ± 0.88 (1)	101.00 ± 3.00 (1)

The positive control used in this study was 4-nitroquinoline 1-oxide (4-NQO) (Sigma) at concentrations of 2 and 1 *μ*g/mL for *S. typhimurium* TA98 and TA100, respectively. His: histidine; DMSO: dimethylsuphoxide (negative control); H_2_O: water (negative control). All cultures were made in triplicate (except the solvent control where five replicates were made). The results are expressed as a mean number of revertants ± standard deviation and mutagenic index values (in parentheses).

## Data Availability

The data used to support the findings of this study are available from the corresponding author upon request.
